# The Engagement of Cytochrome P450 Enzymes in Tryptophan Metabolism

**DOI:** 10.3390/metabo13050629

**Published:** 2023-05-05

**Authors:** Anna Haduch, Ewa Bromek, Wojciech Kuban, Władysława Anna Daniel

**Affiliations:** Department of Pharmacokinetics and Drug Metabolism, Maj Institute of Pharmacology, Polish Academy of Sciences, 31-343 Kraków, Poland; haduch@if-pan.krakow.pl (A.H.); bromek@if-pan.krakow.pl (E.B.); kuban@if-pan.krakow.pl (W.K.)

**Keywords:** cytochrome P450, tryptophan metabolism, serotonin, melatonin, indole metabolites, liver, brain, microbiota, plants

## Abstract

Tryptophan is metabolized along three main metabolic pathways, namely the kynurenine, serotonin and indole pathways. The majority of tryptophan is transformed via the kynurenine pathway, catalyzed by tryptophan-2,3-dioxygenase or indoleamine-2,3-dioxygenase, leading to neuroprotective kynurenic acid or neurotoxic quinolinic acid. Serotonin synthesized by tryptophan hydroxylase, and aromatic L-amino acid decarboxylase enters the metabolic cycle: serotonin → N-acetylserotonin → melatonin → 5-methoxytryptamine→serotonin. Recent studies indicate that serotonin can also be synthesized by cytochrome P450 (CYP), via the CYP2D6-mediated 5-methoxytryptamine O-demethylation, while melatonin is catabolized by CYP1A2, CYP1A1 and CYP1B1 via aromatic 6-hydroxylation and by CYP2C19 and CYP1A2 via O-demethylation. In gut microbes, tryptophan is metabolized to indole and indole derivatives. Some of those metabolites act as activators or inhibitors of the aryl hydrocarbon receptor, thus regulating the expression of CYP1 family enzymes, xenobiotic metabolism and tumorigenesis. The indole formed in this way is further oxidized to indoxyl and indigoid pigments by CYP2A6, CYP2C19 and CYP2E1. The products of gut-microbial tryptophan metabolism can also inhibit the steroid-hormone-synthesizing CYP11A1. In plants, CYP79B2 and CYP79B3 were found to catalyze N-hydroxylation of tryptophan to form indole-3-acetaldoxime while CYP83B1 was reported to form indole-3-acetaldoxime N-oxide in the biosynthetic pathway of indole glucosinolates, considered to be defense compounds and intermediates in the biosynthesis of phytohormones. Thus, cytochrome P450 is engaged in the metabolism of tryptophan and its indole derivatives in humans, animals, plants and microbes, producing biologically active metabolites which exert positive or negative actions on living organisms. Some tryptophan-derived metabolites may influence cytochrome P450 expression, affecting cellular homeostasis and xenobiotic metabolism.

## 1. Introduction

Cytochrome P450 (CYP) is a heme-containing enzyme, the terminal component of the mixed-function oxidase system, catalyzing the oxidative metabolism of endogenous substrates (e.g., steroid hormones) and xenobiotics including drugs, toxins and environmental pollutants [1,2,3]. The cytochrome P450 superfamily is grouped into families and subfamilies according to the evolution process and amino acid sequence identity [4]. Cytochrome P450 is present in humans, animals, plants, fungi, bacteria and viruses [2,5,6,7]. In contrast to eukaryotic organisms whose cytochrome P450 is membrane-bound, bacterial CYP enzymes are soluble in the cytoplasm. The highest amount of cytochrome P450 in humans and animals is found in the liver, but its individual enzymes are present in almost all organs and tissues excluding striated muscle [8].

**Figure 1:**
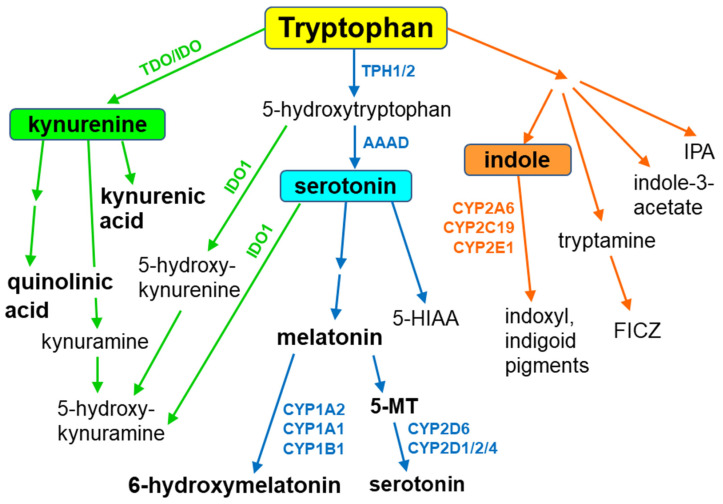
Main pathways of tryptophan metabolism. AAAD = aromatic amino acid decarboxylase; FICZ = 6-formylindolo [3,2-b]carbazole; 5-HIAA = 5-hydroxyindole acetic acid; IDO = indoleamine-2,3-dioxygenase; IPA = indole-3-propionic acid; TPH1/2 = tryptophan hydroxylase 1/2.

Tryptophan, an essential amino acid and a component of different proteins, is metabolized through three main metabolic pathways, namely the kynurenine, serotonin and indole pathways [9] (Figure 1). Tryptophan is not a direct substrate of cytochrome P450 in animals and humans, but it serves as a CYP substrate in plants. However, tryptophan-derived indole metabolites interact with different cytochrome P450 enzymes, yielding biologically active compounds. In addition, in some cytochrome P450 enzyme proteins, tryptophan plays a key role in enzyme survival, as shown for cytochrome P450BM3 (CYP102A1), a bacterial enzyme from *Bacillus megaterium* [10].

The majority of tryptophan is processed via the kynurenine pathway, which is catalyzed by tryptophan-2,3-dioxygenase (TDO, mainly hepatic enzyme) or indoleamine-2,3-dioxygenase (IDO, ubiquitous enzyme), which mediate the formation of kynurenine. Then kynurenine is metabolized in two directions: to the neuroprotective kynurenic acid (an antagonist of α7 nicotinic acetylcholine receptor and of a glycine site in NMDA receptor) and to the neurotoxic NMDA receptor agonist quinolinic acid. Peripheral kynurenine can cross the blood–brain barrier to reach the brain, where, together with locally synthesized kynurenine, it participates in the production of those neuroactive metabolites [11,12]. A potential contribution of cytochrome P450 (CYP) to the kynurenine pathway has not been studied so far.

Serotonin (5-hydroxytryptamine), one of the main monoaminergic neurotransmitters, is synthesized from tryptophan via the classical pathway involving two enzymatic steps: the hydroxylation of tryptophan by tryptophan hydroxylase to 5-hydroxytryptophan and the decarboxylation of 5-hydroxytryptophan by aromatic L-amino acid decarboxylase to serotonin. Serotonin formed in this pathway enters the metabolic cycle: serotonin → N-acetylserotonin → melatonin → 5-methoxytryptamine → serotonin. Recent studies indicate that serotonin can also be synthesized via the alternative pathway involving cytochrome P450, i.e., the CYP2D6-mediated 5-methoxytryptamine O-demethylation in the brain and periphery [13,14,15]. The reaction protects the endogenous indole system, ensuring that its regeneration in the body is maintained in the closed metabolic cycle. However, other cytochrome P450 enzymes (CYP1A1/2, CYP1B1, CYP2C19) are engaged in the catabolism of melatonin [16].

In the gut, approx. 1–2% of dietary tryptophan is metabolized via the tryptophan hydroxylase 1 (TPH1) serotonin pathway, while about 95% of ingested tryptophan is metabolized through the kynurenine pathway. The kynurenine pathway of tryptophan metabolism takes place mainly in the intestinal epithelial cells and antigen-presenting cells. Serotonin can partly enter the kynurenine pathway via biotransformation to 5-hydroxykynuramine by IDO1. 5-Hydroxytryptophan (5-HTP) can also be converted to 5-hydroxykynurenine by IDO1, and then to 5-hydroxykynuramine (reviewed by [17]).

In gut microbes expressing different enzymes, tryptophan is metabolized to indole and indole derivatives. Several bacteria directly convert tryptophan to indole by expressing the enzyme tryptophanase, while other microbes engage various enzymes to produce indole metabolites (indole-3-acetate, tryptamine, indole-3-propionic acid). Some of those metabolites act as activators or inhibitors of the hydrocarbon receptor (AhR), regulating immunity and affecting CYP-catalyzed xenobiotic metabolism [9,18]. The indole formed in this way is further oxidized to form indoxyl and indigoid pigments by CYP2A6, CYP2C19 and CYP2E1 [19,20]. Other products of gut-microbial tryptophan metabolism can inhibit the mitochondrial steroid hormone-synthesizing cytochrome P450 CYP11A1 [21]. Interestingly, aetokthonotoxin, a cyanobacterial neurotoxin that causes vacuolar myelinopathy, consists of a pentabrominated biindole and nitrile group. Recently, the discovery of a productive, five-enzyme biosynthetic pathway was reported, in which two functionalized indole monomers are reunited by biaryl coupling catalyzed by the cytochrome P450 AetB [22].

Apart from the abovementioned indole derivatives, another tryptophan derivative, 6-formylindolo[3,2-b]carbazole (FICZ), has been found as an endogenous ligand mediating AhR signaling and thus regulating homeostatic processes [23]. Being a ligand of an aryl hydrocarbon receptor (AhR) and a substrate of CYP1A1, FICZ is engaged in the FICZ/AhR/CYP1A1 transcriptional–translational feedback loop regulating CYP1A1, CYP1A2, CYP1B1, IL-22 expression and AhR responses, which play a role in immunity, xenobiotic metabolism and tumorigenesis. Cytochrome P450 is also engaged in tryptophan metabolism in plants. CYP79B2 and CYP79B3 were found to catalyze N-hydroxylation of tryptophan to form indole-3-acetaldoxime and CYP83B1 to form indole-3-acetaldoxime N-oxide in the biosynthesis of indole glucosinolates, which are considered to be defense compounds and possibly intermediates in the biosynthesis of phytohormones [24]. On the other hand, thaxtomin phytotoxins (inhibiting cellulose biosynthesis) produced by plant-pathogenic *Streptomyces* species incorporate a nitro group that is essential for phytotoxicity. It was reported that TxtE is a unique new enzyme of the CYP superfamily that catalyzes regiospecific 4-nitration of L-tryptophan utilizing NO and O_2_ [25]. The available literature data indicate that cytochrome P450 is engaged in the metabolism of tryptophan and its indole derivatives in humans, animals, plants and microbes, producing biologically active metabolites of physiological, pharmacological or toxicological importance.

## 2. The Contribution of Cytochrome P450 to the Synthesis of Serotonin

In the brain, serotonin routes originate from neurons of the raphe nuclei, which are located in the brain stem. The dorsal raphe nuclei (DRN) and median raphe nuclei (MRN) neurons project to the forebrain, including the cortex, hippocampus, striatum and hypothalamus, and account for about 80% of forebrain serotonergic endings [26]. Serotonergic projections from the raphe nuclei innervate almost all the brain structures that are involved in controlling important physiological functions, i.e., the cortex (mood and sleep), hippocampus (stress, learning and memory), basal ganglia (motor functions), thalamus (sleep and epilepsy) and hypothalamus (neuroendocrine functions, food intake, circadian rhythm and thermoregulation) [27,28,29]. Thus, serotonin is engaged in the physiology and pathology of various psychiatric disorders and the action of neurological and psychotropic drugs.

**Figure 2:**
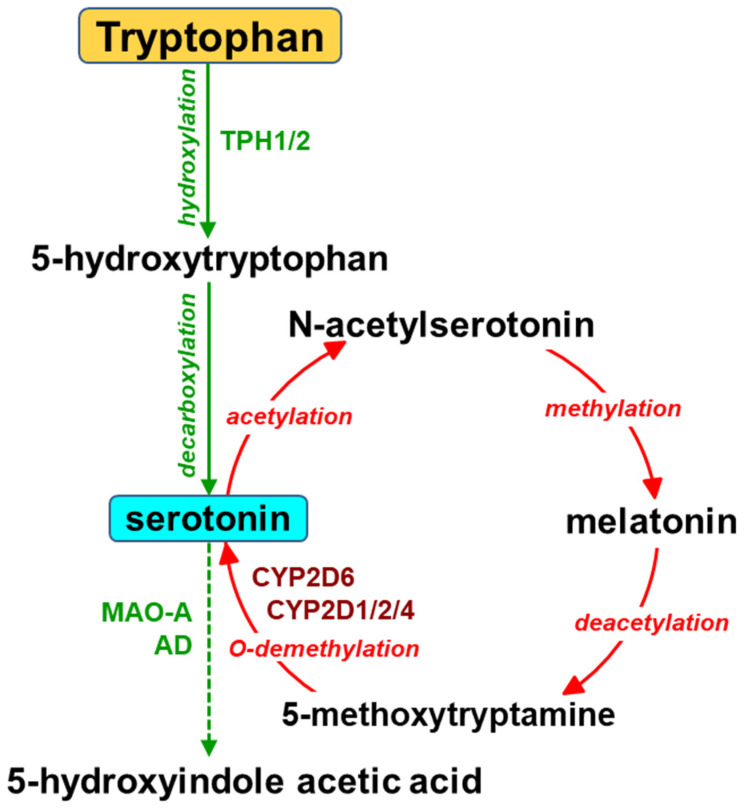
The engagement of cytochrome P450 in the synthesis of serotonin. AD = aldehyde dehydrogenase; MAO-A = monoamine oxidase A; TPH1/2 = tryptophan hydroxylase 1/2.

The biosynthesis of serotonin in the brain starts from the essential amino acid tryptophan and proceeds via hydroxylation to L-5-hydroxytryptophan and subsequent decarboxylation (Figure 2). The activity of tryptophan hydroxylase 2 (TPH2) is considered a concentration-limiting step in the biosynthesis of the neurotransmitter. Serotonin is then inactivated by monoamine oxidase (MAO-A) and aldehyde dehydrogenase to 5-hydroxyindole acetic acid (5-HIAA). Serotonin can also be formed in the gut by tryptophan hydroxylase 1 (TPH1) in enterochromaffin cells and stored in blood platelets. Peripheral serotonin plays an important role in gastrointestinal motility [30,31] and liver regeneration [32,33,34]. It is vitally important that serotonin produced in the periphery cannot penetrate the blood–brain barrier.

Apart from the main (classical) pathway of serotonin synthesis, a possibility of the alternative pathway, i.e., cytochrome P450 2D (CYP2D)-catalyzed O-demethylation of 5-methoxytryptamine to serotonin, has been shown in vitro for human and rat cDNA-expressed CYP2D enzymes and liver microsomes [13,14] as well as for rat brain microsomes [14]. Serotonin formed in this pathway enters the metabolic cycle: serotonin → N-acetylserotonin → melatonin → 5-methoxytryptamine → serotonin (Figure 2). Both melatonin and 5-methoxytryptamine are synthesized in the pineal gland, from which they are released into circulation or to the third brain ventricle. They can also be formed in the periphery (in the gut or the liver, respectively), and then cross the blood–brain barrier. Both brain-derived and liver-derived 5-methoxytryptamine supply a direct substrate for CYP2D enzymes to produce serotonin [35].

Using liver microsomes and cDNA-expressed enzymes, Yu et al. (2003) [13] demonstrated that exclusively human CYP2D6 can catalyze O-demethylation of 5-methoxytryptamine to serotonin. The physiological importance of this reaction in peripheral organs was also shown in vivo by measuring serotonin concentration in the blood plasma of wild-type and CYP2D6-transgenic mice after intravenous injection of 5-methoxytryptamine. CYP2D6-transgenic mice showed a higher plasma concentration of serotonin than CYP2D6-wt mice.

Later studies carried out on rats demonstrated that, of the cDNA-expressed CYP enzymes studied (rat CYP1A1/2, 2A1/2, 2B1, 2C6/11/13, 2D1/2/4/18, 2E1, 3A2), the CYP2D isoforms (CYP2D1, CYP2D2, CYP2D4) were most efficient in catalyzing the O-demethylation of 5-methoxytryptamine to serotonin, though less productive than the human enzyme CYP2D6 [14]. Microsomes obtained from different brain areas (frontal cortex, cortex, hippocampus, thalamus, hypothalamus, brain stem, cerebellum) were able to metabolize 5-methoxytryptamine to serotonin. The highest rate of 5-methoxytryptamine O-demethylation was observed in the brain stem and cerebellum, which are relatively abundant in CYP2D enzyme proteins. The reaction was inhibited by the two specific inhibitors of CYP2D, quinine and fluoxetine [36,37], which proved the selective engagement of CYP2D enzymes in this reaction. The latter studies demonstrated that CYP2D-mediated synthesis of serotonin occurred in the brains of rats and CYP2D6-transgenic mice in vivo [15,38].

The occurrence of 5-methoxytryptamine in concomitance with CYP2D subfamily enzymes in the raphe nuclei pointed to the existence of an additional/alternative pathway of serotonin synthesis [39,40]. Therefore, the exogenous 5-methoxytryptamine was injected into the rostral raphe nuclei (dorsal raphe nuclei, DRN; median raphe nuclei, MRN) comprising serotonin neurons which send their projections to the forebrain [15]. The results obtained after intracerebral administration of 5-methoxytryptamine to male Wistar rats indicated that the formation of serotonin from 5-methoxytryptamine catalyzed by cytochrome P450 might take place in the brain in vivo. The CYP2D-mediated biosynthesis of serotonin was observed by measuring the tissue content of serotonin in different brain regions after 5-methoxytryptamine injection into the rostral raphe nuclei DRN and MRN. The measurements were carried out in naive rats and the tryptophan hydroxylase inhibitor p-chlorphenylalanine (PCPA)-pretreated animals. 5-Methoxytryptamine injected into the rostral raphe nuclei of PCPA-treated rats elevated the tissue concentration of serotonin, while quinine diminished the serotonin level in the cortex and hippocampus of those animals under conditions of partial inhibition of the classical pathway of serotonin synthesis, catalyzed by tryptophan hydroxylase [41].

Serotonin produced by CYP2D enzymes in raphe neurons may move via axonal transport to nerve endings, where it is released into the synaptic cleft. In parallel, neuronal 5-methoxytryptamine may also be transported along axons, being a CYP2D substrate for serotonin synthesis in nerve terminals. Cytochrome P450 is widely distributed within neuronal and glial cells, not only in the endoplasmic reticulum, which is characteristic of the liver, but also in mitochondrial and other cell membrane compartments, in both cell bodies and cell projections [42,43,44]. Considering the above findings, in the next step, the functional extracellular serotonin released from nerve terminals (produced in classical or alternative pathways) was measured by executing the study both in physiological conditions and under the extreme inhibition of tryptophan hydroxylase by PCPA [45]. The functional extracellular neurotransmitter concentration was measured in the frontal cortex and striatum after local intracerebral injection of serotonin, using an in vivo brain microdialysis in male Wistar rats [15]. The probes were implanted in the frontal cortex or striatum, i.e., the brain structures expressing active CYP2D enzymes [42,46] and receiving neuronal projections from serotonin neurons of the rostral raphe nuclei [27]. It is worth noticing that the frontal cortex and striatum are principal brain areas engaged in mood disorders and motor functions, respectively [28]. 5-Methoxytryptamine given locally through a microdialysis probe markedly increased extracellular serotonin levels in the frontal cortex and striatum. Quinine injected jointly with 5-methoxytryptamine prevented the 5-methoxytryptamine-induced increase in cortical serotonin in naive rats and in striatal serotonin in PCPA-treated animals, which testified to the engagement of CYP2D in the serotonin synthesis from 5-methoxytryptamine in vivo [15].

The above-described results obtained in vitro and in vivo in rats [14,15] remain in agreement with those of Cheng et al. (2013) [38], who found a higher level of serotonin and its metabolite 5-HIAA in the brains of *CYP2D6*-transgenic mice than in wild-type animals. Behavioral tests revealed that *CYP2D6*-transgenic mice were also less susceptible to anxiety and depression, which is in line with an important role of serotonin in those mental disorders [47,48,49]. Thus, parallel studies carried out on rats and mice deliver convincing proof that in rodents, serotonin may be synthesized from 5-methoxytryptamine in a CYP2D-catalyzed reaction.

It seems of great interest that the contribution of the alternative pathway of serotonin synthesis is likely to be higher in humans than in rodents since an in vitro study demonstrated that a human CYP2D6 enzyme was appreciably more effective in catalyzing 5-methoxytryptamine O-demethylation to serotonin than rat CYP2D enzymes CYP2D1, CYP2D2 and CYP2D4 [14]. Hence, it may be expected that in the human brain, CYP2D6 has a beneficial effect on the physiological level of active indoleamines, such as serotonin, melatonin and 5-methoxytryptamine. The role of CYP2D6 in the brain may be significant when the classical route of serotonin synthesis governed by tryptophan hydroxylase 2 (TPH2) is impaired [50,51] and/or when the *CYP2D* gene is duplicated or amplified (CYP2D6*2 gene variant) or when CYP2D activity is modified by such inducers as alcohol or nicotine [43,52,53,54,55] or by psychotropic drugs, such as antidepressants [56,57,58,59,60] or neuroleptics [56,61,62,63,64]. The brain serotoninergic system has been shown to be involved in the pathophysiology of psychiatric disorders (depression, anxiety, schizophrenia) as well as in the mechanism of action of psychotropics, including antidepressants, anxiolytics or antipsychotics, respectively. Moreover, it has been observed that individuals with an absent or defective CYP2D6 gene who express a poor metabolizer phenotype are more associable and anxiety-prone [65,66,67], which may be ascribed to a low serotonin level in the brain limbic system [68].

## 3. Melatonin Metabolism by Cytochrome P450

Melatonin is applied in the treatment of sleep disorders, including sleep disturbances accompanying psychiatric diseases such as schizophrenia, depression or seasonal affective disorders [69,70,71,72]. Moreover, its high doses are recommended for neuroprotection [73,74].

The biosynthesis of melatonin in the pineal gland is regulated by the light/dark cycle regulating circadian and circannual rhythms. Moreover, melatonin is also synthesized in a considerable amount in extrapineal organs, in particular in the gastrointestinal tract, where it is synthesized in enterochromaffin cells of the intestinal mucosa and is not controlled by photoperiod [73,74,75]. The synthesis of melatonin from tryptophan through serotonin is catalyzed by N-acetyltransferase and hydroxyindole-O-methyltransferase (Figure 2). The abovementioned two enzymes are expressed in different organs and tissues, including the liver and brain (e.g., in the cortex and striatum), which implies a possibility of melatonin synthesis therein. Extrapineal melatonin is not involved in the regulation of photoperiod, but jointly with pineal melatonin protects cells against the harmful action of oxidative stress due to its antioxidant and anti-inflammatory properties [74]. In addition, melatonin shows anti-excitotoxic activity in the brain by reducing glutamate activity and enhancing that of GABA. Melatonin also stimulates neurogenesis [72,74,75]. As an amphiphilic compound, peripheral melatonin easily penetrates the blood–brain barrier [76].

**Figure 3:**
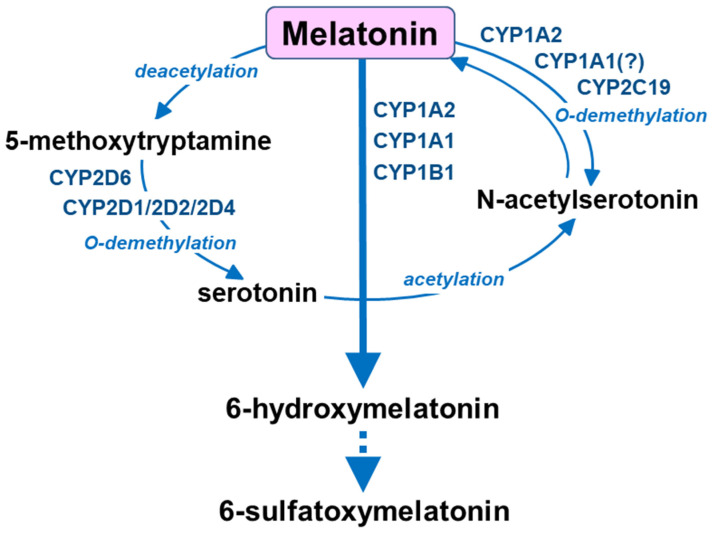
The catabolism of melatonin by cytochrome P450 enzymes.

Compared to serotonin, other cytochrome P450 enzymes are engaged in the catabolism of melatonin: CYP1A2, CYP1A1 and CYP1B1 catalyze aromatic 6-hydroxylation to 6-hydroxymelatonin, while CYP2C19 and CYP1A2 govern O-demethylation to N-acetylserotonin [16,77] (Figure 3). Circulating melatonin is metabolized mainly in the liver by cytochrome P450 enzymes of the CYP1A subfamily (CYP1A1/A2) and CYP2B1 to form 6-hydroxymelatonin, and then conjugate it to 6-sulfatoxymelatonin [16,78,79], but it can also be deacetylated to 5-methoxytryptamine [74,80,81]. In the brain, melatonin and 5-methoxytryptamine are formed mainly in the pineal gland and are then released into circulation or to the third ventricle. On the other hand, 5-methoxytryptamine formed from gut-derived melatonin in the liver penetrates the blood–brain barrier [74,81] and, together with the brain-derived 5-methoxytryptamine, may undergo O-demethylation by brain cytochrome P450 2D (CYP2D) to form serotonin therein. Thus, both endogenous melatonin and exogenously supplied melatonin provide 5-methoxytryptamine in vivo, which may support serotonin formation by the CYP2D-mediated biosynthesis pathway in vivo.

Importantly, Haduch et al. (2016) [82] provided further evidence for the engagement of CYP2D subfamily enzymes in serotonin synthesis in the brain in vivo by demonstrating that exogenous melatonin administered intraperitoneally supported serotonin formation by cytochrome P450 after its deacetylation to 5-methoxytryptamine. Serotonin tissue content in different brain areas (cortex, hippocampus, striatum, nucleus accumbens, thalamus, hypothalamus, brain stem, cerebellum and medulla oblongata) and its extracellular concentration in the striatum were measured (using an in vivo brain microdialysis) after intraperitoneal injection of melatonin to male Wistar rats. Melatonin elevated the tissue concentration of serotonin in the brain areas studied, while the CYP2D inhibitor quinine [36,37] prevented the melatonin-induced increase in serotonin concentration [82]. Melatonin also significantly increased extracellular serotonin levels in the striatum in pargyline-pretreated animals. Pargyline, a monoamine oxidase (MAO) inhibitor, was applied to prevent 5-methoxytryptamine and serotonin oxidation by MAO. In contrast to melatonin, both 5-methoxytryptamine and serotonin are rapidly metabolized by MAO, an enzyme which is abundant in the liver and brain [79,83,84,85]. The CYP2D-inhibitor propafenone injected into the striatum prevented the melatonin-produced rise in striatal serotonin in those animals [82], which testified to the involvement of CYP2D in serotonin elevation observed after melatonin. The above observations confirmed that melatonin supported the CYP2D-catalyzed synthesis of serotonin from 5-methoxytryptamine in the brain in vivo, which closed the serotonin → melatonin → 5-methoxytryptamine → serotonin biochemical cycle. Therefore, it seems that the therapeutic effect of melatonin may result not only from its affinity to melatonin receptors and from its antioxidant properties [72,86] but also from its biotransformation to the neurotransmitter serotonin. The biotransformation of exogenous melatonin to serotonin may be regarded as an additional mechanism of its pharmacological action. These findings are of both physiological and pharmacological importance since melatonin can be synthesized endogenously or administered as a drug for the treatment of sleep and mood disorders as well as a neuroprotective agent [87,88].

## 4. Gut Microbiota Indole Products and Their Interaction with Cytochrome P450

**Figure 4:**
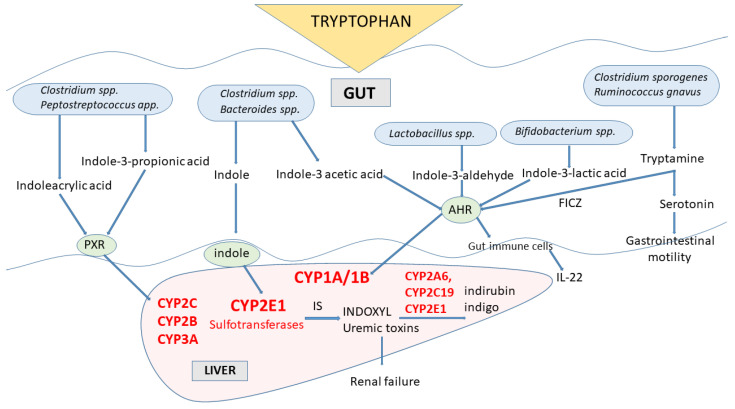
The engagement of cytochrome P450 enzymes in the indole pathway of tryptophan metabolism in microbiota. AHR = aryl hydrocarbon receptor; FICZ = 6-formylindolo[3,2-b]carbazole; IS = indole sulfate; PXR = pregnane X receptor.

In gut microbes expressing different enzymes, tryptophan is metabolized to indole and indole derivatives [89] (Figure 4). Several bacterial species (e.g., *Clostridinum* species and *Bacteroides* species) directly convert tryptophan to indole by expressing the enzyme tryptophanase, while other microbes engage various enzymes to produce a number of tryptophan indole metabolites (e.g., *Clostridium* species, *Peptostreptococcus* species, *Lactobacillus* species, *Bifidobacterium* species). Some of those indole metabolites (tryptamine, skatole, indoleacetic acid, indolealdehyde, indoleacrylic acid, indolelactic acid) act as activators or inhibitors of aryl hydrocarbon receptor (AhR), a cytoplasmic transcription factor that was found in intestinal immune cells, in this way affecting CYP1A/1B subfamily expression and thus xenobiotic metabolism and regulating the IL-22 expression and immune responses [9,18,89]. Indole, indolepropionic acid and indoleacrylic acid influence mucosal homeostasis by decreasing intestinal permeability, possibly mediated by the pregnane X receptor (PXR, a nuclear transcription factor). PXR is one of the nuclear transcription factors engaged in the regulation of cytochrome P450 enzymes’ expression belonging to the subfamilies CYP2C, CYP2B and CYP3A [90]. Tryptophan metabolites migrate through the intestinal epithelium to the blood, where some (indolepropionic acid, indoleethanol, indoleacrylic acid) have antioxidant and anti-inflammatory properties.

The indole formed in the gut microbiota, after being absorbed into the body in substantial amounts, undergoes biotransformation by CYP2E1 in the liver and sulfotransferases to indoxyl-sulfate, which has cytotoxic effects in high concentrations. Indoxyl (3-hydroxyindole) accumulates in patients with chronic kidney disease as a uremic toxin. Further oxidation and dimerization of indoxyl lead to the generation of indigoid pigments indigo and indirubin by CYP2A6, CYP2C19 and CYP2E1 [19,20]. Additional products of indole metabolism by those enzymes were identified as oxindole, isatin, 6-hydroxyindole and dioxindole. The products of gut-microbial tryptophan metabolism, such as indole or skatole, can inhibit the mitochondrial steroid hormone-synthesizing cytochrome P450 CYP11A1 [21], decreasing in this way the synthesis of pregnenolone, which is the precursor of all mineralocorticoids, glucocorticoids and sex steroids. Thus, an excessively tryptophan-rich diet may lead to disrupted steroidogenesis and, in turn, to a decrease in intestinal steroid hormone formation, which negatively influences the course of inflammatory bowel diseases.

The cyanobacterium *Aetokthonos hydrillicola* is expanding on the invasive plant *Hydrilla verticillata*, growing in freshwater lakes. Aetokthonotoxin is a cyanobacterial neurotoxin that causes a fatal neurological disease called vacuolar myelinopathy in birds [91]. The neurotoxin is a structurally configured biindole alkaloid composed of a pentabrominated biindole and nitrile functional group. Recently, an efficient five-enzyme biosynthetic pathway of aetokthonotoxin was described, in which two functionalized tryptophan-derived indole monomers are connected by biaryl coupling catalyzed by the cytochrome P450 AetB [22].

Apart from the abovementioned indole derivatives, another tryptophan derivative, 6-formylindolo[3,2-b] carbazole (FICZ), has been found as an endogenous ligand mediating AhR signaling and thus regulating homeostatic processes [23]. FICZ can be formed either directly from tryptophan (under exposure to light or H_2_O_2_) or from tryptophan metabolites tryptamine (by monoamine oxidase) and indolo-3-pyruvic acid (by indolepyruvate decarboxylase) in microbiota and mammals. Being a high-affinity ligand (Kd = 0.07 nM) of an aryl hydrocarbon receptor (AhR) and a substrate for CYP1A1, FICZ is engaged in the FICZ/AhR/CYP1A1 transcriptional–translational feedback loop regulating CYP1A1, CYP1A2, CYP1B1 and IL-22 expression as well as AhR responses [92,93], which play a role in xenobiotic metabolism, immunity and tumorigenesis. FICZ can influence immune responses depending on its concentration. Low levels of FICZ are pro-inflammatory, developing resistance to pathogenic agents and stimulating antitumor processes, while high FICZ concentrations exert toxicity, cause immune suppression and promote cancer progression. The characterization of tryptophan metabolism and its dysregulation in fibroids revealed an increased expression of CYP1B1 mRNA, a marker of AhR activation [88,94]. ZNF165 (a member of the Kruppel family of zinc-finger-containing transcription factors), which is overexpressed in liver cancer tissues and the immune microenvironment, promotes the proliferation and migration of hepatocellular carcinoma by activating the tryptophan/kynurenine/AhR/CYP1A1 axis and by boosting the expression of CYP1A1 [95].

### Indole Metabolites of Tryptophan-Toxicological Aspects

Several tryptophan metabolites produced by gut microbiota can activate AhR signaling and thus influence different cellular processes. For many of them, biological effects and molecular mechanisms have been proven in in vitro or in vivo tests, including human studies [18,19,20,21,23]. Those active tryptophan-derived AhR agonists can be formed via the kynurenine pathway (kynurenine) or indole pathway (indole, indole sulfate, indole-3-acetic acid, indole-3-aldehyde, tryptamine). Their abnormal activities have been shown in multiple diseases, such as chronic kidney disease, cardiovascular disease, cancer and inflammation (reviewed by [18]). It has been shown that activation of AhR evokes damage to glomerular and tubular cells, which leads to kidney fibrosis. Moreover, activation of AhR stimulates CYP1A1/2 expression, an enzyme engaged in the metabolism of exogenous substrates, including precancerous substances, which results in the production of DNA adducts, causing genotoxicity or induction of oxidative stress and inflammation. Activation of AhR also increases the risk of cardiovascular diseases because of increased expression of cyclooxygenase-2 and, in turn, the accelerated synthesis of prostaglandin and thrombin. This may result in platelet aggregation and vascular dysfunction. Therefore, a tryptophan-balanced diet, controlling gut microbiota composition and providing normal levels of AhR activation and CYP1A1/2 expression are important factors for maintaining homeostasis in health and diseases. Modifying AhR and CYP1A1/2 expression/activity in particular pathological states may be a useful therapeutic strategy.

## 5. The Involvement of Cytochrome P450 in Tryptophan Metabolism in Plants

**Figure 5:**
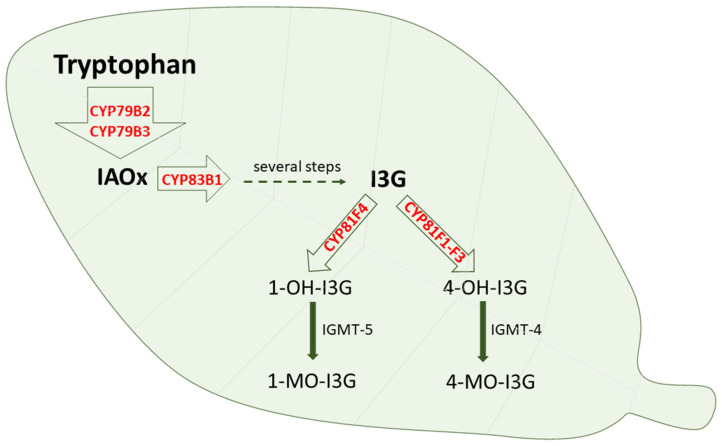
The contribution of cytochrome P450 enzymes to the tryptophan metabolism in plants. IAOx = indole-3-acetaldoxime; I3G = indol-3-ylmethyl glucosinolate; 1-OH-I3G = 1-hydroxy I3G; 4-OH-I3G = 4-hydroxy-I3G; 1-MO-I3G = 1-methoxy-I3G; 4-MO-I3G = 4-methoxy-I3G; IGMT = indole glucosinolate O-methyltransferase.

Cytochrome P450 is also engaged in the oxidative metabolism of endogenous and exogenous substrates in plants and plant microorganisms, where CYP enzymes are present in several organs and organelles, including the endoplasmic reticulum, mitochondria and chloroplasts [96,97]. Plant cytochrome P450 is engaged not only in the metabolism of tryptophan itself but also in the metabolism of tryptophan derivatives. The enzymes CYP79B2 and CYP79B3 were found to catalyze N-hydroxylation of tryptophan to form indole-3-acetaldoxime, while CYP83B1 and CYP83A1 were documented to form indole-3-acetaldoxime N-oxide in the biosynthesis of indole glucosinolates [98]. Glucosinolates are biologically active compounds, possessing anti-insect and anti-plant pathogen defense properties. They are also considered to be possible intermediates in the biosynthesis of the phytohormone indole-3-acetic acid [24,99,100,101,102]. Glucosinolates can be further modified via 4-hydroxylation catalyzed by CYP81F1-F3 or 1-hydroxylation catalyzed by CYP81F4, and the formed hydroxy products undergo O-methylation by O-methyltransferase 5 or 4, respectively [103] (Figure 5). Disruption of the function of the latter enzyme increases the ability to defend against plant pathogens.

In the model plant *Arabidopsis thalia*, cytochrome P450 enzymes contribute to the catabolism of tryptophan, being engaged in many metabolic pathways [104]. Tryptophan is oxidized by CYP79B2 and CYP79B3 to indole-3-acetaldoxime (IAOx). The transcription factors MYB34, MYB51 and MYB122 are regulators of CYP79B2 and CYP79B3 genes, and thus tryptophan metabolism [105]. IAOx is further metabolized by CYP71A12 and CYP1A13 to indole-3-acetonitrile (IAN), and then by CYP71B15 to camalexin. IAN can also be transformed with the participation of CYP71B6 to indole-3-carboxylic acid. Both CYP71A12 and CYP71A13 play an important role in the resistance of *Arabidopsis thalia* against the selected filamentous pathogens *Plectospharella cucumerina* and *Colletotrichum tropicale*; however, CYP71A12 is the major enzyme responsible for the accumulation of indole-3-carboxylic acid in response to pathogens [104]. On the other hand, IAOx is oxidized by CYP83B1 to indole-3-glucosinolates, which contribute to the resistance against *Plectospharella cucumerina* [105]. The abovementioned three secondary indole metabolites of tryptophan glucosinolates, camalexin and indole-3-carboxylic acid are produced by selected cytochromes P450 in response to microbial pathogens and are considered important components of the natural plant immune system.

On the other hand, thaxtomin phytotoxins assembled from L-phenylalanine and L-4-nitrotryptophan are synthesized by plant-pathogenic *Streptomyces* species. They possess a nitro group in a tryptophan moiety that is essential for phytotoxicity caused by inhibiting cellulose biosynthesis. It was reported that TxtE was a unique new member of the CYP superfamily that catalyzed regiospecific 4-nitration of L-tryptophan utilizing NO and O_2_ [25,106,107]. Further studies showed that TxtE had the potential to evolve into a useful aromatic nitration biocatalyst [108].

## 6. Conclusions

The abovementioned discoveries indicate that cytochrome P450 is engaged in the metabolism of tryptophan and its indole derivatives in humans, animals, plants and microbes, producing biologically active metabolites that exert a positive or negative impact on living organisms. On the other hand, some tryptophan-derived metabolites may interact with cytochrome P450 expression, affecting cellular homeostasis, immunity and xenobiotic metabolism. This new knowledge on cytochrome P450 involvement in tryptophan metabolism may be applied to protect organisms against harmful tryptophan metabolites or to develop new pharmacotherapies adopting beneficial outcomes of some tryptophan metabolic pathways, in particular the CYP2D-mediated formation of serotonin in the brain.

It seems that the CYP2D-catalyzed biosynthesis pathway of serotonin synthesis from 5-methoxytryptamine may be recognized as an additional target for the pharmacological action of psychotropic drugs, providing an extra constituent to the already accepted neuronal mechanisms of their medicinal efficacy. The molecular regulation of CYP2D enzymes is not well known, and recent studies indicate that brain CYP2D enzymes are differently regulated compared to their liver counterparts. Moreover, the regulation of brain CYP2D enzymes depends on the studied area. Therefore, the mechanisms of regulation of brain cytochrome P450 enzymes such as human CYP2D6 in particular brain areas should be recognized and specific inducers of enzyme expression developed to enhance serotonin synthesis via alternative pathways in specific brain areas involved in depression.

On the other hand, modifying AhR and CYP1A1/2 expression/activity in particular pathological states may be a useful strategy in the therapy of chronic kidney disease and cardiovascular diseases evoked by toxic tryptophan indole metabolites.

Cytochrome P450 is characterized by genetic polymorphism (the polymorphism of human CYP2D6 is best known). Moreover, the polymorphisms of transcription factors involved in CYP enzyme regulation have also been found (e.g., the polymorphism of AhR regulating CYP1A1/2 and CYP1B1 enzyme expression). Those genetic polymorphisms of CYP proteins may have an impact on the contribution of CYP enzymes to the metabolism of tryptophan, i.e., on the production of serotonin via alternative pathway engaging CYP2D6 or on the AhR/CYP1A/CYP1B1 pathway, toxicity and immune responses.
